# Intact *Staphylococcus* Enterotoxin SEB from Culture Supernatant Detected by MALDI-TOF Mass Spectrometry

**DOI:** 10.3390/toxins11020101

**Published:** 2019-02-09

**Authors:** Jenna Tonacini, Dario Stephan, Guido Vogel, Marc-André Avondet, Franka Kalman, Julien Crovadore, François Lefort, Bruno Schnyder

**Affiliations:** 1 Institute of Life Technologies, University of Applied Sciences, HES-SO Valais/Wallis, Sion 1950, Switzerland; jennatonacini@gmail.com (J.T.); dario.stephan@hevs.ch (D.S.); franka.kalman@hevs.ch (F.K.); 2 Mabritec AG, Food Technology, Riehen 4125, Switzerland; Guido.Vogel@mabritec.com; 3 Spiez Laboratory, Federal Office for Civil Protection, Spiez 3700, Switzerland; Marc-Andre.Avondet@babs.admin.ch; 4 Plants and Pathogens Group, Research Institute Land Nature Environment School of, Architecture and Landscape HEPIA HES-SO University of Applied Sciences and Arts Western Switzerland, 150 route de Presinge, 1254 Geneva/Jussy, Switzerland; julien.crovadore@hesge.ch (J.C.); francois.lefort@hesge.ch (F.L.)

**Keywords:** staphylococcal enterotoxins, superantigen, MALDI-TOF MS

## Abstract

Routine identification of pathogens by MALDI-TOF MS (matrix-assisted laser desorption ionisation time-of-flight mass spectrometry) is based on the fingerprint of intracellular proteins. This work evaluated the use of MALDI-TOF MS for the identification of extracellular pathogen factors. A *Staphylococcus aureus* isolate from a food contaminant was exponentially grown in liquid cultures. Secreted proteins were collected using methanol– chloroform precipitation and analysed by MALDI-TOF MS. A main peak *m*/*z* 28,250 was demonstrated, which was identified as *S.aureus* enterotoxin type B (SEB) by using the pure authentic SEB reference of 28.2 kDa and by amino acid sequence analysis. SEB was also detected in this intact form following pasteurization and cooking treatments. Further application of the elaborated MALDI-TOF MS protocol resulted in the detection of SEA at *m*/*z* 27,032 and SEC at *m*/*z* 27,629. In conclusion, a simple sample preparation from *S.aureus* cultures and an easy-to-perform identification of pathogen factors SE in intact form represents a promising next-generation application of MALDI-TOF MS.

## 1. Introduction

*Staphylococcus aureus* is frequently present on human and animal skin. It is one of the most important causes of chronic, clinical, or subclinical bovine mastitis worldwide. Hygiene-related food contaminations are associated with significant economic losses and can lead to outbreaks of food poisoning. The types and frequency of contaminations vary greatly between countries and can affect processed meats, salads, dairy products, and cooked meals [1,2]. For example, seven of the reported eleven staphylococcal food-poisoning outbreaks from 1994 to 2006 in Switzerland were linked to the consumption of cheese, which is traditionally made from raw milk [3]. The spread of methicillin-resistant *S.aureus* (MRSA) via raw milk cheese is less of a concern than the spread of the intoxicating *Staphylococcus aureus* enterotoxin (SE) [4]. In fact, 74% of foodborne *S.aureus* isolates carry one of the SE genes [4]. SE proteins have superantigen properties in humans, resulting in non-specific polyclonal T-cell expansion followed by a toxic cytokine storm. SEs are capable of inducing emetic intoxications, except for SE-like compounds, comprising one third of the SEs [1,5,6]. The type B SE (SEB) is a prototype SE that has been well characterized and classified as a bioweapon [7]. It is among the twelve most dangerous biological warfare agents, or ‘dirty dozen’, and can easily be aerosolized and is toxic by inhalation.

Emetic foodborne SE intoxications are underdiagnosed to this date for two reasons. Firstly, vomiting reduces doctor’s visits and official registrations. Secondly, the analysis of SE is still incomplete. Twenty-three different SE genes, *sea* to *seu*, have been identified, yet only for a subset of their products (SEA to SEE) are routine immune response tests available [8,9]. This demands a novel approach to identifying SE, a method which may eventually be extended to cover the whole array of SE proteins.

MALDI-TOF MS (matrix-assisted laser desorption ionisation-time-of-flight mass spectrometry) provides a promising approach. Due to its low operational costs and ease of application which does not require a specialist, MALDI-TOF MS has capabilities that rival nucleic acid detection or sequencing. The process is as simple as picking bacterial colonies from an agar plate, loading them on a MALDI TOF target plate, and completing a quick analysis. The bacteria’s proteome fingerprint is identified by searching for a match in databases of reference strains. U.S. and European regulatory agencies have approved these commercial databases composed of ribosomal and other cytoplasmic proteins [10,11,12]. The extracellular proteins released by pathogens are not included in the current databases [13,14], but their inclusion is warranted given the recently published use of MALDI-TOF MS in toxin tests [14,15,16]. Here, SE toxin tests are described. It must be noted that previously published assessments included labor-intensive purification steps prior to MS. Purification of SE type A was performed using SDS-PAGE (sodium dodecyl sulfate polyacrylamide gel electrophoresis) gels and in-gel trypsin fragmentation [16]. Alternatively, the toxins were also affinity-purified using antibodies bound to magnetic beads [15]. In order to establish an easy-to-use routine test, a simple sample preparation process without the need for laborious purifications was required.

The objective of our study was to improve the MALDI-TOF MS test of SE by evaluating a rapid sample preparation of the intact form, which allows a fingerprint identification similar to pathogen identification.

## 2. Results

### 2.1. Analysis of *S.aureus* Using Established MALDI-TOF MS Methods

**Figure 1:**
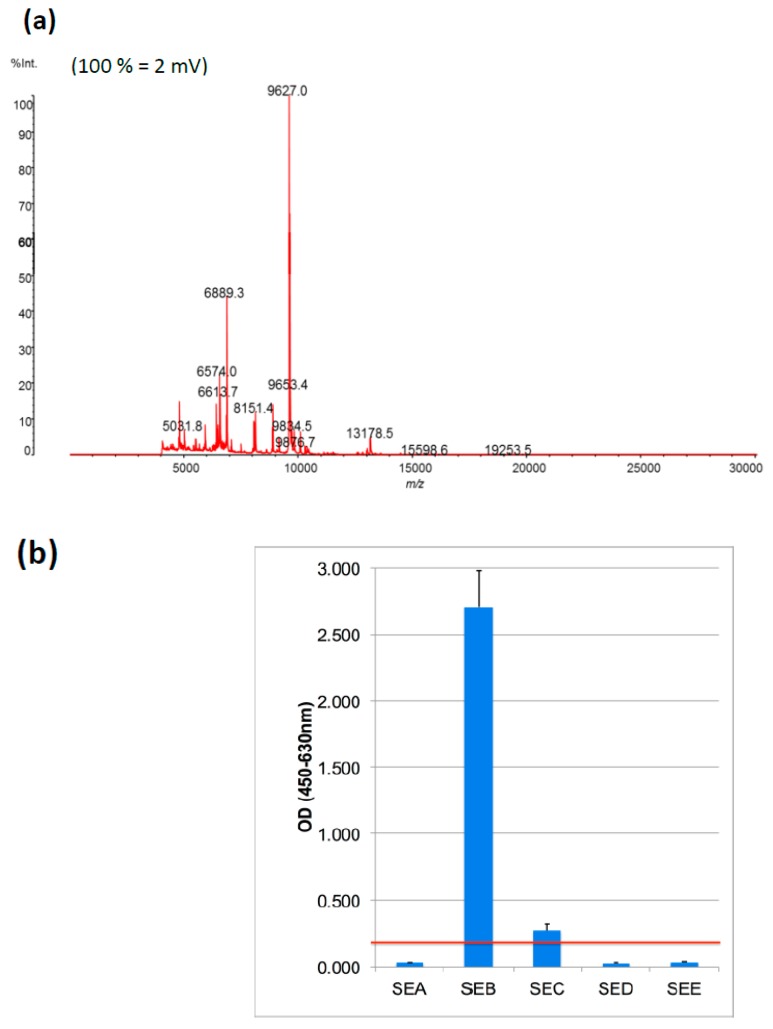
(**a**) Matrix-assisted laser desorption ionisation time-of-flight mass spectrometry (MALDI-TOF) mass spectra of intracellular proteins between *m*/*z* 4000 and *m*/*z* 30,000 (indicated on x-axis and on top of the peaks) of *S.aureus* strain CCM5757, grown at 37 °C for 48 h, is shown. Peak intensity arbitrary units as provided by the *LaunchPad* program is given on the y-axis. (**b**) Proteins secreted by exponentially grown *S.aureus* CCM5757 were analysed using an immune-based qualitative analysis of *S. aureus* enterotoxins SEA to SEE (ELISA), as indicated on the x-axis. The y-axis (OD_450_nm) indicates the detection signal of the enzyme-linked color reaction, with the red line determining the threshold level and SE positivity (above the line). The columns show mean +/− SD of five technical replicates.

The protein profile of a smear of a bacterial isolate (strain CCM5757) of interest was studied using MALDI-TOF MS. The spectrum profile of cytoplasmic proteins (*m*/*z* 3,000 to *m*/*z* 20,000) identified the smear as *S.aureus* (Figure 1a), matching with the Saramis database and confirming its original description.

The bacteria were separated from the cell-free part (supernatant) by centrifugation. The removal of bacteria prevented MS-spectra saturation by the abundant cytoplasmic proteins. Secreted proteins in the supernatant were first analyzed by established methods before assessment with novel MS-based methods.

The commercially available immune assay (ELISA) detected the prominent presence of SE toxin. Figure 1b shows that SE type B (SEB) cumulated in the culture supernatant of *S.aureus* CCM5757.

For the novel analysis, using MALDI-TOF MS, the secreted proteins underwent first an SE sample preparation as described in the following. SE proteins were successfully precipitated from the culture supernatant of *S.aureus* CCM5757 using methanol/chloroform (described in detail in the methods section).

Protein precipitation by isoelectric precipitation or by trichloroacetic acid (TCA) precipitation resulted in unsuccessful SE sample preparation for the MALDI-TOF MS analysis. Such unsuccessful or absent SE signals were compared with the *S.aureus* strain DSM799, the SE-negative control.

### 2.2. Detection of *S.aureus* Toxin SEB Using MALDI-TOF MS

**Figure 2:**
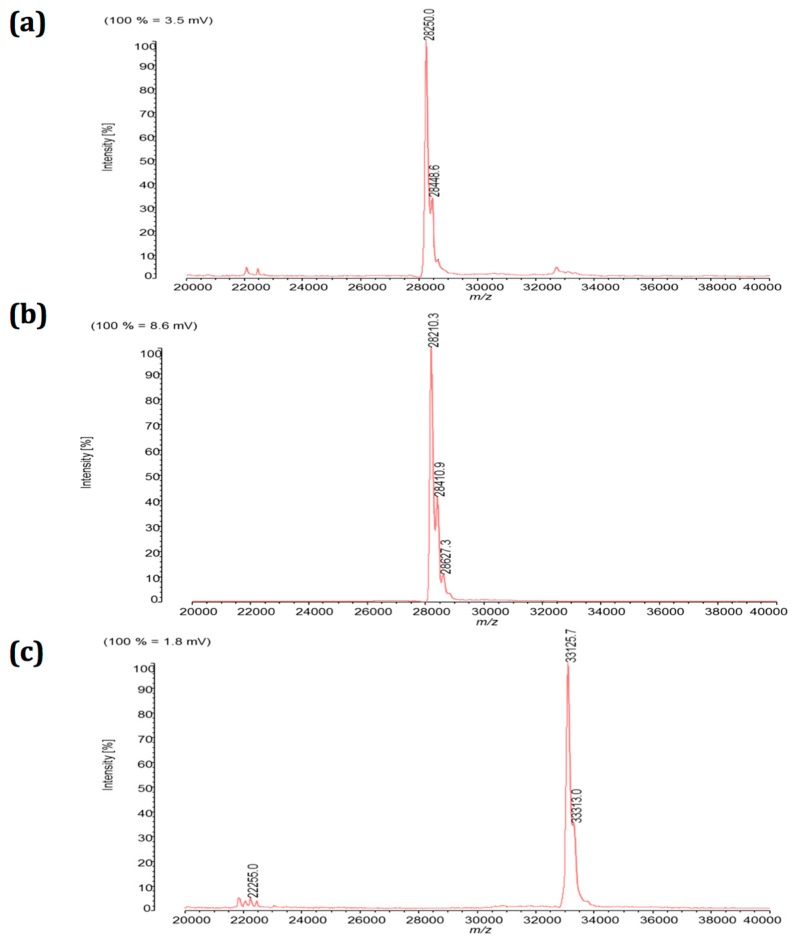
MALDI-TOF mass spectra of extracellular proteins between *m*/*z* 20,000 and *m*/*z* 40,000 (indicated on x-axis) are shown with the respective peak intensities (in percentage of maximal, given on y-axis). Following sample preparation, SEB was detected either (**a**) in unpurified form from supernatants of *S.aureus* strain CCM5757, or (**b**) in purified form from commercially available reference proteins (100 µg/mL). (**c**) *S.aureus* reference strain DSM799 lacking the SEB gene was analysed in parallel as negative control. Figures are representative for at least five independent experiments.

One µl of the prepared *S.aureus* supernatant was spotted on the MALDI target plate. The minute-lasting MALDI-TOF MS resulted in a main MS peak *m*/*z* 28,250 (Figure 2a). The peak corresponded to the purified reference SEB of *m*/*z* 28,210 (Figure 2b). When reference SEB was diluted in a decimal dilution series, it was detected at concentrations as low as 0.1 µg/mL. Thus, a MALDI-TOF MS protocol was established, which demonstrated the molecular mass of freshly secreted SEB in its intact and virtually functional form. 

A closer look at the SEB signal in Figure 2a shows a major *m*/*z* 28,250 peak and a minor *m*/*z* 28,448 peak. The double peak was shown for the *S.aureus* supernatant derived SEB (Figure 2a) and the commercial SEB (Figure 2b). The identification of the minor peak needs further investigations in future studies but may partially be illuminated by a recent study [17]. The SEB double peak may have formed as a result of different post-translational modifications, truncations by proteolytic degradations, or most likely by different signal peptide cleavages. Gene variations of *seb* as recently found in different *S.aureus* strains, however, do not seem to be the reason for the double peak found in a given *S.aureus* strain [18]. Signal peptides are removed, resulting in the release of mature proteins from the microbe. In fact, signal peptide processing can occur at more than one site of SE toxins [17], which virtually causes different sizes of secreted toxins and eventually the double peak MS signal in Figure 2.

Figure 2c further shows that a second virulence factor was secreted. From *S.aureus* DSM799 supernatant, a single MS peak *m*/*z* 33,130 was identified. This value correlated to the published value of α-haemolysin of 33,176 Da. α-Haemolysin was absent from the SE-positive *S.aureus* CCM5757 strain (Figure 2b), which is in agreement with previous findings that expression and secretion of α-haemolysin is not strictly linked to the expression of SE [19]. In addition to strain CCM5757, *S.aureus* strain SA-022 also resulted in an SEB signal using both methods, ELISA and MALDI-TOF MS.

### 2.3. Consolidation of the SEB Signal by Fragmentation and Amino Acid Sequencing

**Figure 3:**
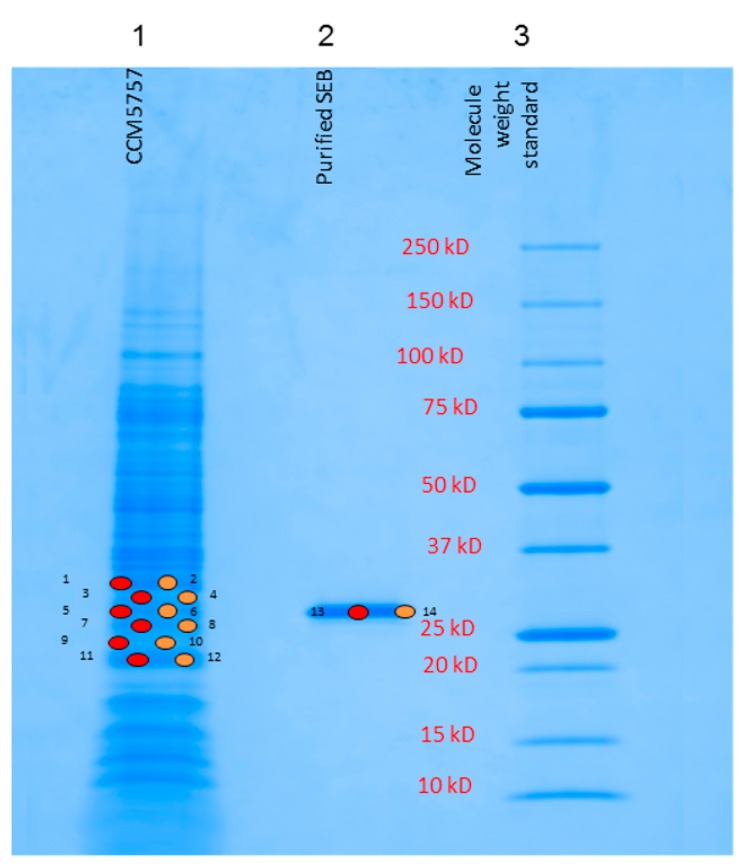
SDS-PAGE electrophoresis using 4–20% gels separated the *Staphylococcus* supernatant proteins. Protein bands were stained by Coomassie blue, sampled at indicated points, and analysed by microsequencing. Lane 1 on the gel shows secreted proteins from *S.aureus* strain CCM5757, lane 2 shows purified SEB (Sigma, Buchs, Switzerland) in the range of 28.2 kDa.

To validate the detection of the 28.25 kDa protein in the MALDI-TOF MS spectra, its amino acid sequence was determined. The *S.aureus* strain CCM5757 was grown exponentially in culture, and the cell-freed supernatant was processed for SEB precipitation. Upon resuspension of the precipitate, it was separated by SDS-PAGE electrophoresis on a precast 4–20% gel (Figure 3). Coomassie blue-stained protein bands migrating at 28.2 kDa were excised and digested by trypsin. The SE fragments were microsequenced using LC (liquid chromatography)/MS–MS and identified according to the published database Mascot. SEB was successfully identified with a 47% protein coverage and with an ion score of 527. This was similar to purified reference SEB sequencing with a 46% protein coverage and an ion score of 1534. Therefore, the trypsin digestion of SEB followed by protein microsequencing confirms the identity of the full length or intact SEB toxin detected by MALDI-TOF MS. The *S.aureus* supernatant protein of 33,176 Da was identified by microsequencing as α-haemolysin with a 42% protein coverage and with an ion score of 1118.

By comparing the two distinct analyses of the same *S.aureus* culture supernatant, the SDS-PAGE analysis (Figure 3) can detect a vast array of secretome proteins, while the MALDI-TOF MS (Figure 2a) provides a small selection of signals, restricted to those proteins which have been ionized by the MALDI matrix. The SE toxins belong to the abundant MS signals. Yet, there are smaller peaks present in the SE toxin sample. However, they remain minor likely because an MS-spectra saturation by the abundant signals has occurred. 

### 2.4. Limits and Promises of the SE Test Protocol

The test was applied to heat-treated SEB. SEB was treated at 70 °C and 95 °C for 5 min or was treated at 70 °C and 95 °C for 30 min. The MS signal was not altered in its molecular weight of 28.3 kDa for any of the heat treatments compared to untreated samples. Therefore, successful detection of SEB following pasteurization and near-cooking heat treatments further established the application of MALDI-TOF MS for toxin testing.

In contrast to the successful SEB protocol, using *S.aureus* laboratory cultures, as shown above, the direct analysis of SEB in the foodstuff matrix shows limitations. Milk was spiked with 1 mL of *S.aureus* CCM5757 supernatant containing SEB. The milk samples consisted 9 mL of 10% (*v*/*v*), 1%, 0.1%, or 0% pasteurized milk. In the 1%, 0.1%, and 0% milk matrices, SEB was detected as the characteristic MS peak (Figure S1). In the presence of 10% milk matrix, the background signals of the MS spectra were significantly elevated. They virtually saturated the MS spectra and allowed no detection of SEB (signal-to-noise, 3:1). Similar results were obtained when testing SEB in cheese matrix instead of milk. Therefore, concentrated food matrices such as the classical 1/10 dilution (10%) of a food sample affects the sensitivity of the established SEB test, which in turn restrains the SEB test application to *S.aureus* laboratory cultures.

**Figure 4:**
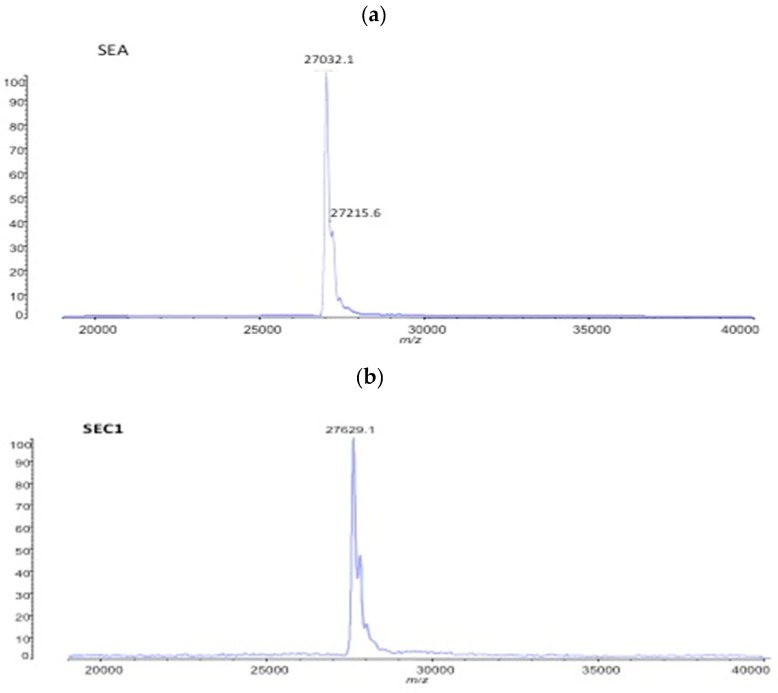
(**a**) MALDI-TOF mass spectra of purified toxin SEA (100 µg/mL) and (**b**) SEC1 (100 µg/mL), shown with the respective peak intensities (in percentage of maximal, given on y-axis). Figures are representative for at least three independent experiments.

The SE test was further applied to enterotoxins SEA and SEC. Their authentic references were detected at the intact size of SEA at *m*/*z* 27,032 and at the intact size of SEC at *m*/*z* 27,629 (Figure 4). The peak *m*/*z* 27,032 corresponds to 27.1 kDa for SEA [20] and *m*/*z* 27,629 corresponds to 27.53 kDa for SEC [21]. These findings show that the identification of an array of SE, including SEA to SEC, is obtained by the here-established MALDI-TOF MS analysis.

## 3. Discussion

The MALDI-TOF MS described in this study provides a new application by successfully identifying secreted pathogen factors. The MS peak at *m*/*z* 28,250 represented the 28.3 kDa *Staphylococcus* enterotoxin B [22]. The *m*/*z* 33,130 peak represented the 33.2 kDa α-haemolysin. Both are important virulence factors released by *S.aureus*. Hence, the SEB test proposed in this study identifies SEB toxin in its intact and likely functional form. This presents significant advantages over genetic tests of *seb* because these signals do not guarantee real production of the toxin.

The production of SEB was confirmed through complementary identifications. We selected the *S.aureus* strain CCM5757 according to the published presence of the *seb* gene [23]. The presence of SEB was proven by using the commercial method of immune-based ELISA. The MALDI-TOF MS peak of supernatant SEB was identified by its mass position at the size of pure standard SEB. Finally, enzyme-digested fragmentation spectra were comparable with the theoretical expectations, and their amino acid sequence analysis validated the SEB identification.

The extracellular SEB was prepared using methanol/chloroform precipitation. The methanol/chloroform precipitation removed cytosolic remains of *S.aureus* in the sample. A subsequent addition of proton-donating MALDI-TOF matrix enabled us for the first time to detect SEB toxin as a dominant and ionizable (protonized) protein fingerprint at 28.25 kDa (Figure 2). So far, MALDI-TOF MS has been authorized for germ identification using clinical and food-contaminating microbe isolates [24]. Our extracellular fingerprinting complements this process. While *S.aureus* bacteria are identified by their dominant and ionizable protein profile of ribosomes and the cytosol sized between 4 and 12 kDa, larger proteins typical of extracellular pathogen factors can now be detected, as described for the 28.25 kDa SEB (Figure 1).

The protocol of using MALDI-TOF MS for secreted SEB provides several advantages. Previous studies identified fragments, rather than intact forms, of the SEA and SEB toxins by MALDI-TOF MS or LC-MS/MS [16,25]. Their method was laborious due to the need for trypsin digestion of SE into fragments of 0.8 to 2.4 kDa, and it was thus relevant mainly only for research purposes. For example, we applied the trypsin-digestion sample preparation for the identification of SEB by sequence analysis. The sample preparation of fragments of SE was significantly longer (several hours) and more laborious than the sample preparation of intact SE using a simple precipitation (completed within minutes). The protocol of MALDI-TOF MS is also advantageous with respect to the commercial ELISA analysis of SE due to its low operational costs.

The MALDI-TOF MS of SE has a further advantage over the commercial ELISA. In Figure 1, the antibody-based ELISA test shows the presence of SEB as well as a second reproducible signal suggesting SEC presence as well. Our genome analysis [23] ascribed the *seb* gene, but not *sec*, to the corresponding *S.aureus* strain. This indicates inaccurate ELISA results. Indeed, the provider’s instructions caution about 20% cross-reactivity of SEB antibodies with SEC. The established MALDI-TOF MS analysis, however, is accurate. In Figure 2, an SEB signal was selectively found in the respective *S.aureus* sample. Since the SEB peak *m*/*z* 28,300 is distinguishable from SEC (*m*/*z* 27,600) a false positive signal was hence excluded when using MALDI-TOF MS. 

Further promising SE test systems show that immune, PCR-based analysis of SE is remarkably sensitive [26]. However, it is also dependent on the quality of the SE antibodies. The best antibody quality is achieved when the above described cross-reactivity of antibodies between SEB and SEC as well as between SEA and SEE are eliminated. Such quality improvement virtually is ambitious as all SE toxins share up to three conserved motive structures and risk sharing antibody reactivity (cross-reactivity) [5]. In contrast, the sizes of the various SE toxins are different, enabling MALDI-TOF MS to accurately distinguish them. MALDI-TOF MS even bears the potential of a non-targeted test system. Various SE toxins as well as α-haemolysin can be detected. Previous studies have described the respective identification of *S.aureus* delta toxin and leukocidin [13,14].

The antibody tests of SE are described and normed for detection of the toxin in the food matrix. This is particularly advantageous when the resistant toxins but no more living *S.aureus* are present in the intoxicated food. The established MALDI-TOF MS analysis, although detecting SEB in milk and cheese, lost sensitivity due to food matrix effects. Yet, MALDI-TOF MS analysis of SE toxins remains promising, for example, for testing food contaminations with remaining live *S.aureus*. Live pathogen identification by using the normed MALDI-TOF MS can be improved by adding information on the risk of emesis and superantigen presence. 

The entire food chain, from primary products to processed and packaged products, can now be controlled routinely for intoxications. On identification of an *S.aureus* isolate by MALDI-TOF MS based on a colony smear on agar plates, exponential growth cultures of the same isolate are run under agitation [27]. Following this quick culture supernatant sample preparation, identification of SEB by MALDI-TOF MS is completed within less than a minute, the whole process taking only a few minutes. In conclusion, MALDI-TOF MS can be used to detect secreted pathogen factors in their intact size. It is a rapid and precise method which shows promise for future routine tests of microbial toxins.

## 4. Materials and Methods

### 4.1. MALDI-TOF MS Analysis of *S.aureus* Isolates

*S.aureus* strain CCM5757 is also known as ATCC 14458 (and FDA 243), and strain DSM 799 as ATCC 6538 (and FDA 209). Strain SA-022 is a foodborne isolate whose identity as *S.aureus* has previously been published [23]. 

*S.aureus* strains isolated from food or clinical samples were analyzed by MALDI-TOF mass spectrometry. For each isolate, one colony grown on agar plates was selected, resuspended in 1 mL of 100% ethanol solution, precipitated (5 min, 16,200 RCF (relative centrifugal force)), and resuspended in 60 µL of 70% formic acid and 100% acetonitrile (at a 1:1 ratio). After a second centrifugation, 1 µL of the supernatant was transferred to the MALDI-TOF stainless steel target plate and was dried. One ul of the MALDI matrix, sinapic acid, was added, and the sample was analyzed by MALDI-TOF MS (*Shimadzu*) within a minute. The protein fingerprint was identified in a database according to the SARAMIS (Spectral ARchiving and Microbial Identification System) program. Strains were identified by a coverage of 98% or higher when compared with database *S.aureus* isolates.

Alternatively, bacteria samples were applied on the MALDI stainless steel target plates using a sterile inoculation loop. One µL of matrix (α-cyano-4-hydroxycinnamic acid) was added and allowed to crystallize for 10 min. Then, the MALDI-TOF mass spectrometer identified the sample as described above.

### 4.2. MALDI-TOF MS Analysis of Secreted SE Proteins

One to ten millilitres of *S.aureus* culture supernatants originating from cell cultures grown for 48 h in TSB (tryptic soy broth) at 37 °C, or otherwise as indicated, were precipitated using the methanol/chloroform method (protein/methanol/chloroform/H_2_O at ratio of 1/4/1/3) [28]. Three phases were observed from bottom to top, namely the chloroform phase containing phospholipid components, the intermediate phase with precipitated proteins, and the methanol phase containing mainly the particles and whole cells. The latter overlaying phase was discarded. The intermediate phase was complemented with >3 volumes of H_2_O, was centrifuged at 17,000 RCF for 1 min, and the protein disc between the 2 phases was freed from the upper layer. Then, methanol (300 µL) was added, was centrifuged at 10,000 RCF for 4 min, and the pellet was dried out.

The pellet was resuspended in formic acid (10 µL of a 5% solution). The formic acid sample solution was diluted fivefold in a sinapic acid matrix, and 1 µL was transferred to the MALDI-TOF stainless steel target plate. The sample was dried and analysed by the MALDI-TOF MS. Ion gate, pulsation number and laser intensity vary between routine identification of the bacterium and the secreted-protein analysis by MALDI-TOF MS. The sample was dried and analysed by the MALDI-TOF MS using the Axima Confidence instrument from Shimadzu Biotech in the linear positive mode. The protein mass spectra were acquired and compared using the Shimadzu *LaunchPad 2.8* program [11]. The mass range for measuring SEA, SEB, and SEC was set to 19–70 kDa with the ion gate at 18 kDa. One-hundred profiles with 10 laser shots each were acquired automatically on 100 raster points separated by a distance of 120 µm. The pulsed extraction was set to 28,000, and the laser power was adjusted to obtain optimal signal intensities and resolutions depending on the state of the laser. The calibration of the instrument was performed with bovine serum albumin using the one-, two-, and threefold protonated signals. 

The MALDI-TOF MS analysis provides the precise m/z mass-to-charge ratio indications of each protein peak (on x-axis) and the peak intensity values on the y-axis of the figures, indicating either the presence or absence of a peak (voltage forces applied are indicated in the figures). Authentic SEA standard was provided by Sigma, SEB, SEC by Toxin Technology (Sarasota, FL, USA).

### 4.3. Heat Treatments of SE Toxin

*S.aureus* strain CCM5757 was cultured for 48 h at 37 °C, then agitated at 150 rpm. Supernatants were treated at 70 °C and 95 °C for either 5 min or for 30 min and agitated at 200 rpm. This was followed by SE sample preparation (using methanol/chloroform) and analysis by MALDI-TOF MS of secreted SE proteins, as described above.

### 4.4. Analysis of SE in the Foodstuff Matrix Milk

*S.aureus* CCM5757 supernatants originating from 48-h cell cultures grown in TSB at 37 °C were spiked in a volume of 1 mL to the following food sample preparations. Different concentrations of pasteurized milk, originally containing 2.5% of fat, were each spiked by the 1-mL SEB-containing supernatant. The sample suspension was precipitated by the methanol/chloroform method and analysed by MALDI-TOF MS of secreted SE proteins, as described above. This sample preparation protocol of foodborne SEB was optimal, and no laborious steps like isoelectric precipitation for the removal of casein proteins of milk were included.

### 4.5. SEB Protein Sequencing Using LC-MS–MS

*Staphylococcus* supernatants of 48-h grown cell cultures were precipitated using the methanol/chloroform method (protein/methanol/chloroform/H_2_O at ratio of 1/4/1/3), as described above. Following centrifugation at 17,000 RCF, the precipitate was suspended in phosphate-buffered saline (PBS) pH 7.4 (1/5 of original volume). The protein solution was separated on a precast 4–20% SDS-PAGE gel electrophoresis (*BioRad*, Cressier, Switzerland). The Coomassie-blue-stained protein band at 28.2 kDa was excised, washed with an ammonium hydrogen carbonate buffer and acetonitrile. The proteins in the band were digested with trypsin (20 µg/mL) at pH 8 for 180 min at 37 °C. The protein fragments were microsequenced using LC-MS–MS as follows and identified according to the MASCOT database. Following the trypsin digestion, the peptides were desalted and dissolved in 0.2% formic acid for LC-MS/MS analysis. They were separated at 600 nL/min flow rate for 30 minutes (linear gradient 5–40% acetonitrile/0.2% formic acid) on a C18 reversed-phase nLC system. Full MS in the orbitrap (Thermo; 300–1400 *m*/*z*) was acquired at 60,000 resolution, AGC 5e5, and maximal injection time of 100 ms. For tandem MS scan events, the resolution was set to 30,000, AGC to 5e4, 27% HCD collision energy, and a maximum injection time of 120 ms.

### 4.6. ELISA-Based Analysis of Secreted SE Proteins

Antibody-based ELISA analyses of SE toxins from *S.aureus* cell culture supernatant were performed according to the instructions by the manufacturer (R-biopharm, Dramstadt, Germany) ridascreen SET A,B,C,D,E).

## Supplementary Materials

The following are available online at https://www.mdpi.com/2072-6651/11/2/101/s1, Figure S1: Milk solutions containing 10%, 1%, 0.1%, or 0% of food matrix (indicated in the figure) were each spiked with equal amounts of unpurified SEB from *S.aureus*. Following sample preparation, the proteins were analysed focusing on the identification of the 28.21 to 28.26 kDa SEB protein. The MALDI-TOF mass spectra show the fingerprints of extracellular proteins between *m*/*z* 20,000 and *m*/*z* 40,000 (indicated on the x-axis and on top of the peaks) and their arbitrary peak intensities given on the y-axis. Figures are representative for at least 3 independent experiments.
