# Supplementary material for: Intact Staphylococcus Enterotoxin SEB from Culture Supernatant Detected by MALDI-TOF Mass Spectrometry
Source: Toxins (Basel). 2019 Feb 9;11(2):101. doi: 10.3390/toxins11020101 (PMC6409910; doi:10.3390/toxins11020101)
Supplement: Supplementary file 1 [file toxins-11-00101-s001.pdf]

Supplementary Materials: Intact *Staphylococcus* Enterotoxin SEB from Culture Supernatant Detected by MALDI-TOF Mass Spectrometry

Jenna Tonacini, Dario Stephan, Guido Vogel, Marc-André Avondet, Franka Kalman, Julien Crovadore, François Lefort and Bruno Schnyder

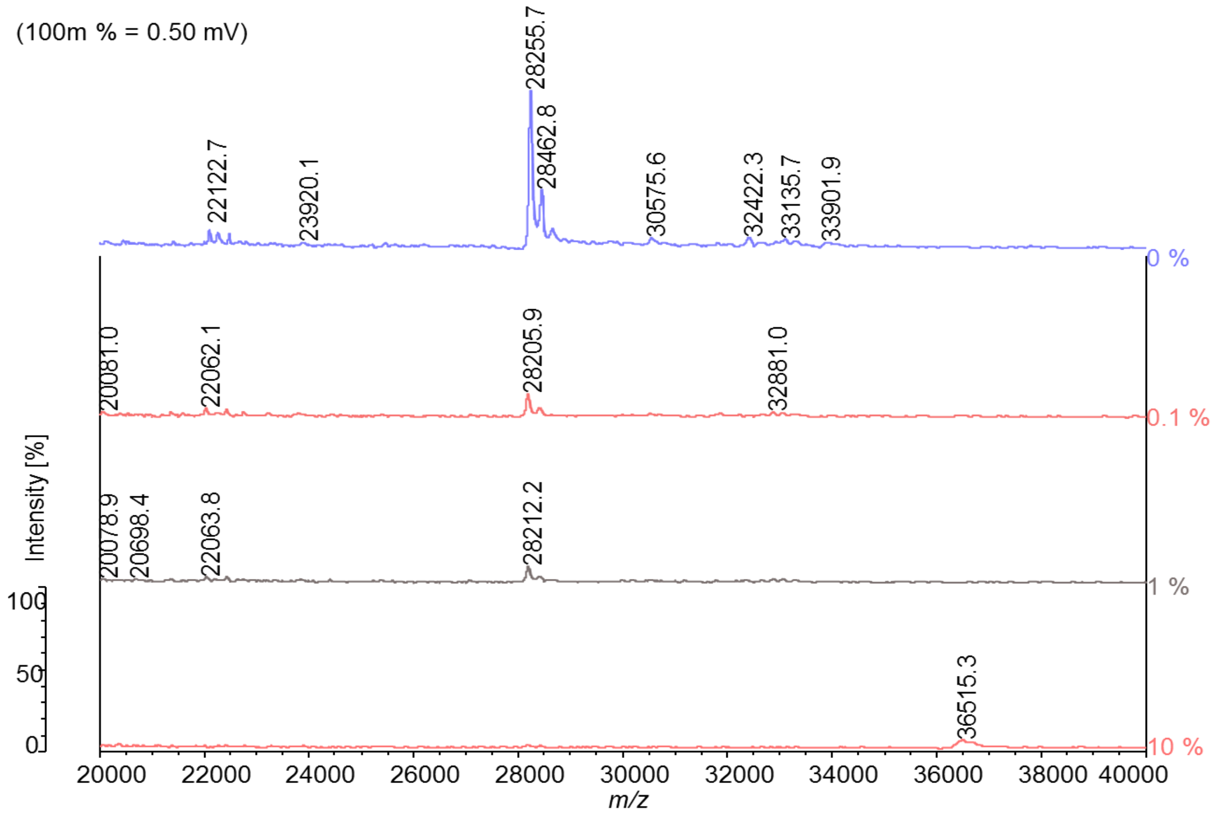

Figure 1. Milk solutions containing 10%, 1%, 0.1%, or 0% of food matrix (indicated in the figure) were each spiked with equal amounts of unpurified SEB from *S.aureus*. Following sample preparation, the proteins were analysed focusing on the identification of the 28.21 to 28.26 kDa SEB protein. The MALDI-TOF mass spectra show the fingerprints of extracellular proteins between *m*/*z* 20,000 and *m*/*z* 40,000 (indicated on the x-axis and on top of the peaks) and their arbitrary peak intensities given on the y-axis. Figures are representative for at least 3 independent experiments.
